# Thrombosis Risk History and D-dimer Levels in Asymptomatic Individuals with Prader–Willi Syndrome

**DOI:** 10.3390/jcm11072040

**Published:** 2022-04-05

**Authors:** Lisa Matesevac, Jennifer L. Miller, Shawn E. McCandless, Jaret L. Malloy, Jessica E. Bohonowych, Caroline Vrana-Diaz, Theresa V. Strong

**Affiliations:** 1 Foundation for Prader–Willi Research, Walnut, CA 91789, USA; lisa.matesevac@fpwr.org (L.M.); jessica.bohonowych@fpwr.org (J.E.B.); caroline@fpwr.org (C.V.-D.); 2 Department of Pediatrics, College of Medicine, University of Florida, Gainesville, FL 32611, USA; millejl@peds.ufl.edu; 3 Department of Pediatrics, University of Colorado Anschutz Medical Campus, Aurora, CO 80045, USA; shawn.mccandless@childrenscolorado.org; 4 JLM Clin Dev Consulting, LLC, San Diego, CA 92120, USA; jlmclindev@gmail.com

**Keywords:** Prader–Willi syndrome, thrombosis, D-dimer, blood clot

## Abstract

Individuals with Prader–Willi syndrome (PWS) may be at higher risk of developing blood clots as compared to the typical population, but this risk is poorly understood. It is also unclear if laboratory testing of D-dimer concentration might be useful to screen for thrombosis in PWS. Here, we surveyed the thrombosis history of 883 individuals with PWS and evaluated the D-dimer concentration in a subset of 214 asymptomatic individuals, ages 5–55. A history of at least one blood clot was reported by 3.6% of respondents. Thrombosis increased with age, but no significant difference was found on the basis of sex or family history. Genetic subtype was a significant factor when considering only those with a known subtype, and individuals with a history of edema had significantly more blood clots. In the D-dimer sub-study, ≈15% of participants had high D-dimer concentrations, and 3.7% had D-dimer values more than twice the normal upper limit. One participant with a high D-dimer result was found to have a blood clot. No significant differences in D-dimer results were found on the basis of age, sex, genetic subtype, family history of blood clots, edema history, or BMI. The D-dimer test does not appear to be a sensitive and specific screening tool for blood clots in asymptomatic individuals with PWS.

## 1. Introduction

Prader–Willi syndrome (PWS) is a rare neurodevelopmental disorder with an estimated incidence of 1 in 10,000 to 30,000 live births [1,2,3]. PWS is caused by loss of expression of paternally expressed, imprinted genes within the region of chromosome 15q11.2-q13. There are three main genetic subtypes of PWS. Approximately 60% of individuals with PWS have a deletion in the paternal chromosome 15, ≈35% have maternal uniparental disomy (UPD) (both chromosome 15s are maternally inherited), and 3–5% have an imprinting defect (paternal chromosome 15 with a maternal imprint). Rarely, PWS is caused by chromosomal abnormalities such as translocation or inversion [4]. The syndrome is characterized in early years by hypotonia and failure to thrive, followed later by the onset of hyperphagia (excessive appetite), a hallmark feature of the syndrome. In the absence of strict environmental controls, individuals with PWS will become morbidly obese. Additional characteristics of the syndrome include short stature, cognitive disability, endocrine abnormalities, hypogonadism, sleep disturbances, and a challenging neurobehavioral phenotype [3,4].

The mortality rate for individuals with PWS is ≥3 times higher than the normal population and has been estimated at 1–4% per year, with the mean age of death in the third decade [5,6,7]. The causes of death for individuals with PWS vary and are often related to the consequences of morbid obesity. However, additional risk factors may be present in the PWS population. Pulmonary embolism (PE) is one of the leading causes of reported death in the PWS population, with at least 7% of deaths attributable to this cause as reported in a 40-year mortality study [6,7].

Following two fatal events of pulmonary embolism in obese adults in the context of a PWS clinical trial evaluating the investigational drug beloranib for hyperphagia and weight loss [8], Manzardo, et al. reported venous thromboembolism events (VTEs) as a previously unrecognized significant medical risk in the PWS population, even in the absence of experimental drug use [9]. Survey data from over 1000 PWS families indicated that VTEs occurred at a rate of 3–4% of individuals with PWS [9], significantly higher than what is reported for the general population (<1%) [10]. Data from the Danish Health Registries further supports an increased prevalence of VTEs in individuals with PWS versus an age- and sex-matched non-PWS comparison group, although the PWS group was more likely to be obese and have diabetes than the comparison group [11]. However, a review of insurance health claims found the occurrence of VTEs in obese individuals with PWS was 2.55 times higher than in an obese, non-PWS control group [12]. The incidence of VTEs within the PWS population is correlated with co-morbidities including obesity, edema, and metabolic syndrome [9]. The occurrence of VTEs in individuals with PWS also increases with age, consistent with the general population [10], and is likely due to increased prevalence of co-morbidities with age. Notably, PWS genetic subtype has not been shown to be a predictor of thrombosis risk [9].

Given the high rate of VTEs in PWS, a screening tool to identify those at highest risk is an important goal. D-dimer represents the smallest fibrinolysis-specific degradation fragment present in the circulation as a blood clot dissolves. Laboratory measurement of circulating D-dimer concentration is routinely used to exclude a diagnosis of deep vein thrombosis (DVT) or PE [13]. We sought to better define the rate of thrombotic events in individuals with PWS and evaluate the potential utility of D-dimer screening to inform the prevalence of thrombotic events.

## 2. Materials and Methods

**Thrombosis History Survey.** The thrombosis history survey was completed by the caregiver (respondent) of the person with PWS (participant), with data collected in the Global PWS Registry [14]. All protocols and surveys were reviewed and approved by the New England IRB (NEIRB), and all legal guardians/legally authorized representatives of the person with PWS completed an informed consent. The study population included participants with PWS for which the survey was completed between September 2018 and January 2022. Data were collected regarding personal history of blood clots, biological family history of blood clots, age at which blood clot occurred, location in the body, and edema history, among other items.

**D-dimer Determination**. Enrollment in the D-dimer study began in October 2018 as a sub-study of the *Paving the way for Advances in Treatments and Health for PWS (PATH for PWS)* study (NCT03718416), which is a prospective natural history study of PWS designed to better understand the serious medical events occurring in the population, as well as changes in PWS behaviors over time. Upon enrolling in the *PATH for PWS* natural history study, legal guardians/legally authorized representatives of participants located in the United States could elect to consent to participate in the D-dimer sub-study, which had a target enrollment of 200. Once enrolled, a requisition for a LabCorp blood draw was generated using the city and state of the participant. All blood draws were completed at the participant’s local LabCorp location, and the results were returned to the respondent and to the study coordinator through a secure website. If the result of the testing was outside of the normal range, the participant’s caregiver/guardian was contacted to review the findings and risk factors for blood clots by a national clinician network contracted with the study, PWNHealth. Each respondent was advised to seek medical guidance from the participant’s primary physician regarding the abnormal findings, and a copy of the results were provided to the respondent for reference. If the value exceeded the normal range by more than twice the normal limit, in addition to seeking medical guidance, respondents were asked to take the participant to have a repeat blood draw completed. These participants were provided a new requisition to complete the repeat testing. All respondents of study participants were provided a copy of the results for their records.

**Data Analysis** Descriptive statistics are presented (proportions and means with standard deviations). Chi-squared tests, Fisher’s exact tests, and t-tests were used for bivariate analysis (Social Science Statistics online calculator). All statistical tests were performed with a two-tailed α level of 0.05.

## 3. Results

### 3.1. Thrombosis History Survey Results

**Figure 1:**
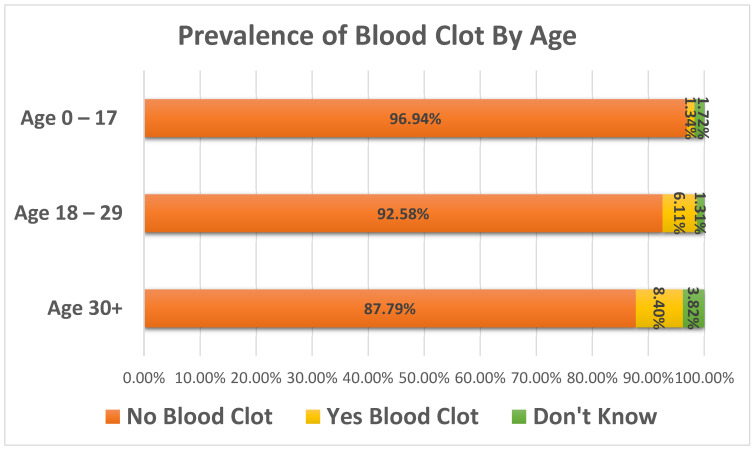
Prevalence of blood clots by age group in PWS participants who completed the Thrombosis History Survey. Respondents were asked whether the participant (i.e., the person with PWS) had ever had a blood clot. The age distribution of those participants over the age of 30 was 83 individuals aged 30–39, 35 individuals aged 40–49, 12 individuals aged 50–59, and 1 individual aged 60+.

A total of 883 responses obtained in the *Thrombosis History* survey were analyzed with respect to overall incidence of blood clots, age of first blood clot, location of blood clots, incidence of blood clots by PWS subtype, family history of blood clots, and edema history. Gender representation among survey responses was approximately equal, with 52.4% of the participants reported as female and 47.6% reported as male. The PWS subtypes of the group were 49% Deletion, 32.3% UPD, 2.8% Imprinting Defect, and 15.9% were reported as Other/Unknown/Unreported. Of the total number of responses, 3.6% (n = 32) reported the person with PWS as having a history of one or more blood clots. Prevalence of blood clots varied significantly by age group (Figure 1), with 1.3% (7/523) of 0–17-year-olds, 6.1% (14/229) of 18–29-year-olds, and 8.4% (11/131) of 30+ year-olds (age range 30–61) reporting ever having a blood clot (X^2^ = 23.7, *p* < 0.0001).

**Figure 2:**
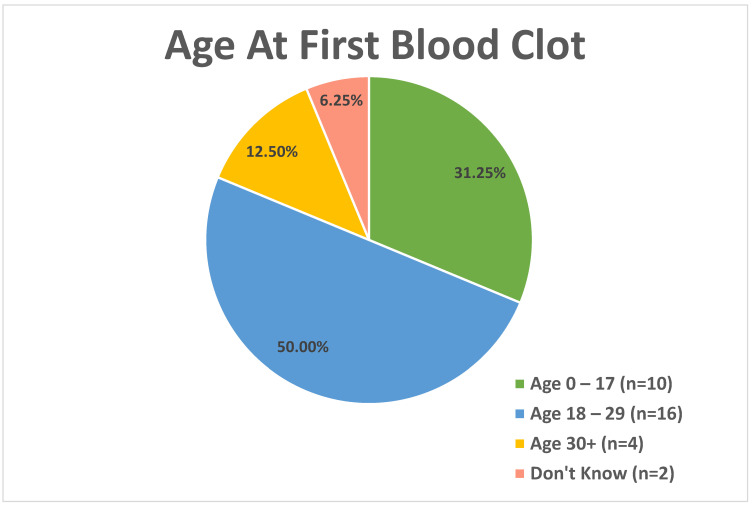
Age at first blood clot in PWS participants who reported a blood clot in the Thrombosis History Survey.

For those reporting a positive history of blood clot, age at first blood clot was analyzed in three age categories (Figure 2), with 31.3% (n = 10) reporting the first blood clot between the ages of 0 and 17, 50% (n = 16) in the 18–29 age group, 12.5% (n = 4) in the 30+ age group, and 6.3% (n = 2) reporting that they do not know the age at which the first blood clot occurred. The average age at first blood clot was 20.4 years of age (SD: 12.5 years).

**Figure 3:**
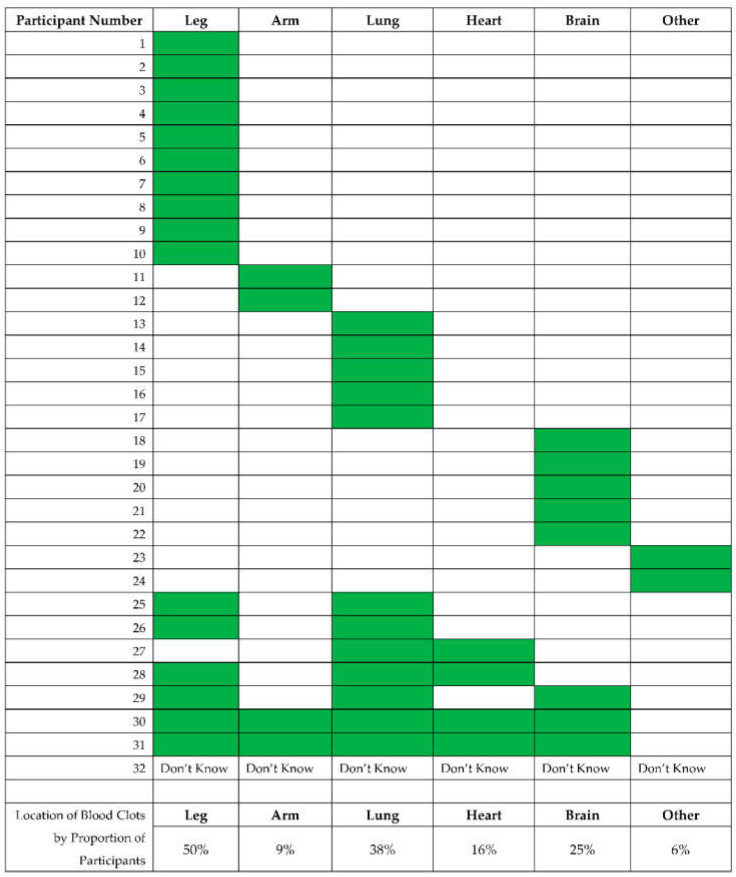
Location of blood clots in 32 PWS participants who reported a blood clot in the Thrombosis History Survey. Participants were able to indicate if they had blood clots in more than one location, and participants could also report a single clot or multiple clots in each location.

Respondents were asked to document the area of the body in which the thrombotic event occurred. A total of 75% of individuals experienced one or more blood clots in a single location, primarily in extremities such as the arms and legs. However, some participants have experienced clots in more than one part of the body (Figure 3).

A significant association was found between prevalence of blood clots and history of edema. Of those reporting a history of edema, 10% (10/100) had a blood clot, while 2.5% (19/747) of those without a history of edema had a blood clot, and 8.3% (3/36) of those with an unknown previous edema history had a blood clot (X^2^ = 92.9, *p* < 0.0001). There was no statistically significant difference in prevalence of blood clots by family history (first degree relatives) of blood clots. Of those with a family history of blood clots, 5.4% (6/111) had a blood clot, while 3.1% (22/716) of those with no family history of blood clots had a blood clot, and 7.1% (4/56) of those with an unknown family history had a blood clot (X^2^ = 5.1, *p* = 0.27).

Participants in the Global PWS Registry also completed a *Diagnosis* survey, in which participants indicate the genetic subtype of PWS, if known. There was a higher rate of blood clots reported in individuals with PWS due to chromosome deletion, but this did not reach statistical significance compared to other groups (among deletion subtype 4.9% (21/433) recorded blood clots, among UPD subtype 1.4% (4/285) recorded blood clots, 0% (0/25) were recorded in the Imprinting subtype, and 5% recorded blood clots in the “Other”/Don’t Know group (7/140); *p* = 0.096). However, when only comparing individuals with known genetic subtypes, individuals with PWS due to deletion did have a statistically higher rate of blood clots compared to the other genetic subtypes (Deletion: 4.9%, UPD: 1.4%, Imprinting: 0%, Fisher’s exact test; *p* = 0.029). The number of participants who do not know their subtype may be attributed to the large number of older participants for whom subtype specific testing was not available at the time of diagnosis, or in whom the diagnosis was made on the basis of clinical presentation of symptoms. There was no statistically significant difference in prevalence of blood clots by gender; 3.45% (16/463) of females had a blood clot and 3.8% (16/420) of males had a blood clot.

### 3.2. D-dimer Sub-Study

A total of 318 *PATH for PWS* participants enrolled and consented to participate in the D-dimer sub-study. Of those, 215 participants completed the blood draw. One participant was excluded due to the sample being of insufficient quality. Of those who completed the blood test, 32.7% (n = 70) participants were in the 5–11-year-old age range, 29.4% (n = 63) participants were in the 12–17-year-old age range, and 37.9% (n = 81) participants were in the 18+ year-old age range. Distribution by sex was equal, with 108 male participants and 105 female participants. The genetic subtype distribution of the participants was 53.9% reporting PWS deletion subtype (n = 115), 33.8% reporting PWS UPD subtype (n = 72), 0.9% reporting PWS Imprinting defect subtype (n = 2), 0.9% reporting PWS via chromosomal translocation (n = 2), and 10.3% reporting an unknown PWS type (n = 22).

**Figure 4:**
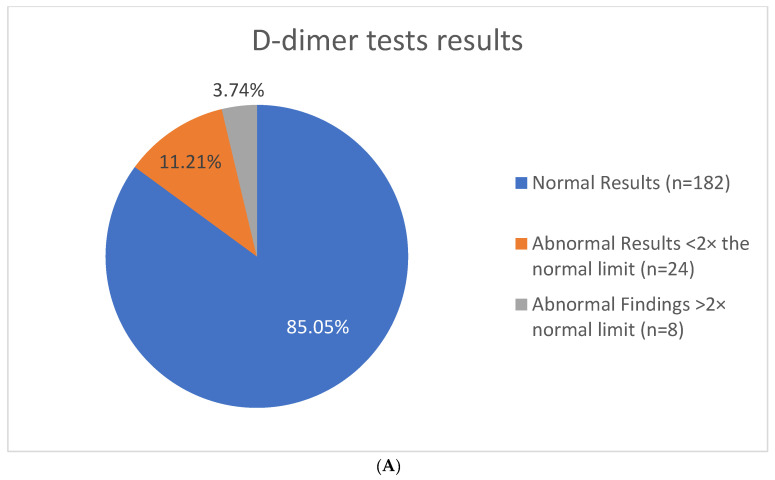

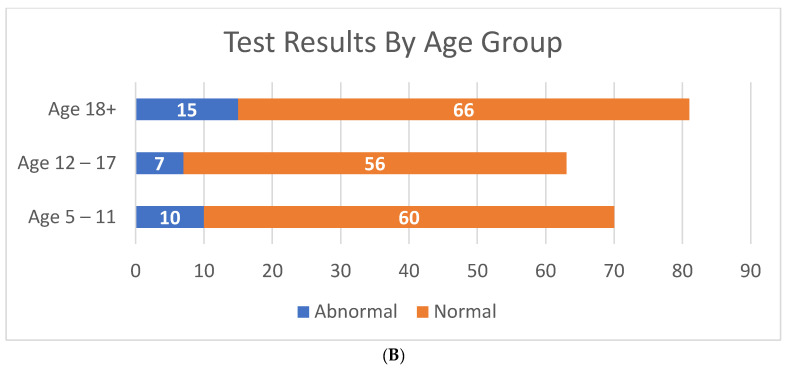
Laboratory findings of D-dimer concentration in 214 individuals with PWS. (**A**) Overall results showing number of participants with abnormal D-dimer findings. (**B**) Distribution of abnormal D-dimer results in different age categories.

Results of D-dimer testing are available for 214 participants (Figure 4A). LabCorp reference range was established as 0.00 to 0.49 mg/L FEU. Of the completed tests, 14.95% (n = 32) had D-dimer values that exceeded the reference range. Within the different age brackets, 14.3% (n = 10) of children aged 5–11 years old had abnormal results, while 11.1% (n = 7) in the 12–17 years old age group and 18.5% (n = 15) of those in the 18+ age group were above the reference range (Figure 4B).

Of the abnormal findings, 3.73% (n = 8) results exceeded the normal reference range by more than two times the normal limit. Repeat testing was completed on seven of the eight subjects, and 71.45% (n = 5) had abnormal findings upon repeat testing, but no clinically detected blood clots were reported in this group. Of the total number of participants with an abnormal result, one was discovered to have a blood clot. The D-dimer concentrations of this individual were modestly elevated (<2× normal limit). The participant’s caregiver was advised to follow up with the participant’s primary care physician for evaluation, and a blood clot in her leg was diagnosed upon physical exam and ultrasound testing. The participant, who was obese and in her early 20s, was prescribed a Factor Xa inhibitor.

The proportion of D-dimer abnormal test results did not differ significantly by age, sex, genetic subtype, BMI, family history of blood clots, or family history of edema (Table 1). Analysis with respect to race and ethnicity was not conducted as 85% of participants reported as White, making analysis based on this metric inconclusive.

## 4. Discussion

Individuals with PWS have a greater risk for VTEs such as PE and deep vein thrombosis compared to their age-matched peers with a diagnosis of obesity. Further, the incidence of obesity in PWS is a risk factor for increased incidence of VTEs [9,11,12]. Therefore, a sensitive and specific screening test for risk of VTEs would be of particular value for this population. This study examined VTE risk factors in a large, registry-based PWS population and evaluated the utility of D-dimer testing in asymptomatic individuals with PWS.

Thrombosis history data from 883 individuals in the Global PWS Registry showed that prevalence of blood clots increased with age, with 8% of individuals with PWS above the age of 30 reporting at least one blood clot. In addition, there was a statistically significant difference in prevalence of blood clots associated with edema, with a higher prevalence of blood clots among those reporting a history of edema compared to those with no previous edema history or unknown edema history. Edema and age were the only two covariates ascertained that were significantly associated with increased blood clots in this analysis. The prevalence of blood clots in individuals with PWS reported here (3.6%) is slightly higher than a previous study documenting a 3% incidence of thrombosis in a cohort of 1067 individuals with PWS, noting that there is likely some overlap among participants between the two cohorts [9]. Consistent with these data, that study similarly showed clot location more commonly found in the extremities, as well as no correlation between blood clots and gender or PWS genetic subtype. However, the previous study did find a correlation between blood clots and those with a family history of blood clots, which was not found here.

The incidence of blood clots in the general population is estimated at 1:100,000 to 1:100 depending upon, and increasing with, age. The maximum estimated risk is ≈1% in the non-PWS population 80 years and older [10]. Thus, the findings reported here, with 8% of individuals with PWS age 30 and above reporting blood clots, is significantly higher than that in the general population, particularly noting that no individuals in this study were over the age of 65. In addition, the age of onset of blood clots is younger in the PWS as compared to the non-PWS population. In this study, 80% of individuals who have had a blood clot experienced the first clot by age 29, consistent with the report by Manzardo et al., with a mean age of clot identification of 32.8 years [9]. This differs from the general population in which the risk of DVT/PE rises slowly until age 50 and then begins to increase substantially, disproportionally affecting the elderly [10].

That there was no difference in prevalence of blood clots in PWS by gender in this and prior studies is also in contrast to the non-PWS population, where females of child-bearing age are at greater risk than males in the same age group, shifting to a greater risk in males in the over 50 age group [10]. This is attributed to gender differences in the hormone levels influential in clot formation. Our failure to identify similar gender differences in PWS cases may be related to disorder-specific neuroendocrine disruption, with associated hypogonadism and sexual immaturity that is characteristic of PWS. A limitation of the current study is that the retrospective nature of thrombotic event reporting did not allow detailed collection of data regarding concomitant medication use, gonadal status, or BMI at the time of the event, limiting the ability to draw associations between these factors and blood clot occurrence.

To better understand the potential for a standard laboratory test to inform VTE risk in this rare disease, we evaluated D-dimer concentrations in asymptomatic individuals with PWS. As a rare disease with a dispersed population, a decentralized approach of recruiting through electronic outreach/social media and local blood draws allowed the study to be completed in a time- and cost-efficient manner. The D-dimer sub-study recruitment was completed over a period of 9 months, and D-dimer blood draws for 215 participants was concluded in the 11th month from study inception. Of the completed D-dimer tests, almost 15% had abnormally elevated D-dimer concentrations, which were reported to the study respondent. Caregivers of participants with abnormal results were advised to consult with the primary care physician, and those with abnormal results more than two times the normal range were additionally advised to have repeat D-dimer testing. Some of those with an abnormal test result proceeded to have additional evaluations as ordered by their primary care physician, including ultrasound and/or echocardiogram. Of those with abnormal results, one participant was found to have a blood clot (1 of 32 participants with high D-dimer values, a 3% yield).

The rate of abnormal findings of D-dimer concentration did not correlate well with the incidence of diagnosed blood clots in this study. No statistically significant differences in elevated D-dimer levels were found on the basis of sex, genetic subtype, family history of blood clots, edema history, or BMI. Additionally, there was no significant increase in abnormal findings on the basis of age in this cohort, as might have been expected. Pieper et al. showed a significant correlation of high D-dimer levels with age in an elderly cohort, with D-dimer levels increasing 25.9% with each decade of advancing age [15]. A 2013 meta-analysis suggests that age adjusted cut off values for D-dimer concentrations would improve the clinical utility of D-dimer testing in people greater than 50 years of age and result in decreased false positive results. Specificity of D-dimer testing in patients under 50 years of age suspected of having venous thromboembolism is reported as 49–67% and is as low as 0–18% in individuals over the age of 80 years [16]. A retrospective study of D-dimer testing in children suggests use of D-dimer testing can exclude PE in children with a low probability of PE who demonstrate normal D-dimer levels. A sensitivity of 100% and specificity of 58% was documented [17]. A retrospective study reported that D-dimer concentrations in children were significantly elevated in the presence of a VTE compared to those without VTE [18]. However, another study showed children with PE were as likely to have a normal D-dimer value as those without PE [19]. These studies show that there is variability in the reported potential of D-dimer as a tool for detecting blood clot risk.

As for the general population, this study of individuals with PWS suggests that D-dimer concentrations are unlikely to be an informative tool for predicting individualized risk for VTEs in the asymptomatic PWS population. This study was not designed to determine the diagnostic value of D-dimer measurements in people with PWS presenting with symptoms suggestive of VTE; however, this may be a more informative use of this assay. In addition, in certain clinical settings, D-dimer concentrations have been shown to have some potential utility in monitoring VTE recurrence [20,21]. Additional studies are needed to determine if, in the setting of PWS patients with a history of a previous blood clot or a documented history of edema, D-dimer concentrations may have some clinical value in informing the risk of blood clot incidence.

## Figures and Tables

**Table 1:** Bivariate analysis of D-dimer test results by sociodemographic characteristics.

Initial D-dimer Results			
Sociodemographics, N(%)	Abnormal	Normal	Total
Age, mean (SD)	18.41 (10.38)	16.40 (9.12)	16.7 (9.32)
Gender			
Male	14 (13.0)	94 (87.0)	108 (50.7)
Female	18 (17.0)	88 (83.0)	106 (49.5)
Genetic Subtype			
Deletion	14 (12.0)	103 (88.0)	117 (54.7)
UPD	14 (19.4)	58 (80.6)	72 (33.6)
Imprinting defect	1 (50)	1 (50)	2 (0.9)
Translocation	0 (0)	2 (100)	2 (0.9)
Don’t know	3 (14.3)	18 (85.7)	21 (9.8)
Deletion Subtype			
Type I (large)	3 (20)	12 (80)	15 (13.2)
Type II (small)	1 (3.2)	30 (96.7)	31 (27.2)
Other	1 (25)	3 (75)	4 (3.5)
Don’t know	9 (14.1)	55 (85.9)	64 (56.1)
BMI, mean (SD)	28.9 (9.88)	26.4 (9.66)	26.7 (9.71)
BMI Category			
Underweight	0 (0)	1 (100)	1 (0.5)
Normal weight	8 (10.1)	71 (89.9)	79 (37.4)
Overweight	5 (12.5)	35 (87.5)	40 (19.0)
Obese	19 (20.9)	72 (79.1)	91 (43.1)
Family History of Blood Clots			
Yes family history	7 (22.6)	24 (77.4)	31 (14.5)
No family history	24 (14.6)	141 (85.4)	165 (77.1)
Don’t know family history	1 (5.6)	17 (94.4)	18 (8.4)
Family History of Edema			
Yes family history	6 (28.6)	15 (71.4)	21 (9.8)
No family history	25 (13.4)	162 (86.6)	187 (87.4)
Don’t know family history	1 (16.7)	5 (83.3)	6 (2.8)

Note: No bivariate analysis was statistically significant with chi-squared tests.

## Data Availability

The data presented in this study are available on request from the corresponding author.
